# Age‐dependent expression of cancer‐related genes in a long‐lived seabird

**DOI:** 10.1111/eva.13024

**Published:** 2020-06-15

**Authors:** Richard Meitern, Jérôme Fort, Mathieu Giraudeau, Kalev Rattiste, Elin Sild, Tuul Sepp

**Affiliations:** ^1^ Institute of Ecology and Earth Sciences University of Tartu Tartu Estonia; ^2^ Littoral Environnement et Sociétés (LIENSs) UMR 7266 CNRS‐La Rochelle Université La Rochelle France; ^3^ CREEC/CREES MIVEGEC, UMR IRD/CNRS/UM 5290 Montpellier France; ^4^ Institute of Agricultural and Environmental Sciences Estonian University of Life Sciences Tartu Estonia

**Keywords:** cancer defenses, gene expression, *Larus canus*, senescence, transcriptome, wild animals

## Abstract

Studies of model animals like mice and rats have led to great advances in our understanding of the process of tumorigenesis, but this line of study has less to offer for understanding the mechanisms of cancer resistance. Increasing the diversity of nonmodel species from the perspective of molecular mechanisms of natural cancer resistance can lead to new insights into the evolution of protective mechanisms against neoplastic processes and to a wider understanding of natural cancer defense mechanisms. Such knowledge could then eventually be harnessed for the development of human cancer therapies. We suggest here that seabirds are promising, albeit currently completely ignored candidates for studying cancer defense mechanisms, as they have a longer maximum life span than expected from their body size and rates of energy metabolism and may have thus evolved mechanisms to limit neoplasia progression, especially at older ages. We here apply a novel, intraspecific approach of comparing old and young seabirds for improving our understanding of aging and neoplastic processes in natural settings. We used the long‐lived common gulls (*Larus canus*) for studying the age‐related pattern of expression of cancer‐related genes, based on transcriptome analysis and databases of orthologues of human cancer genes. The analysis of differently expressed cancer‐related genes between young and old gulls indicated that similarly to humans, age is potentially affecting cancer risk in this species. Out of eleven differentially expressed cancer‐related genes between the groups, three were likely artifactually linked to cancer. The remaining eight were downregulated in old gulls compared to young ones. The downregulation of five of them could be interpreted as a mechanism suppressing neoplasia risk and three as increasing the risk. Based on these results, we suggest that old gulls differ from young ones both from the aspect of cancer susceptibility and tumor suppression at the genetic level.

## 1 INTRODUCTION

Malignant, “selfish” cells affect all multicellular organisms (Giraudeau, Sepp, Ujvari, Ewald, & Thomas, 2018; Leroi, Koufopanou, & Burt, 2003; Madsen et al., 2017; Roche, Møller, Degregori, & Thomas, 2017). They can lead to the life‐threatening disease of cancer for which we still know too little about in ecological settings. They can also act as a selection pressure, as physiological mechanisms aimed at fighting malignant cells likely come with trade‐offs with other functions (Thomas et al., 2017). It is increasingly demonstrated that oncogenic phenomena, from precancerous lesions to final stages called metastatic cancers, are extremely prevalent in host populations, and not just in postreproductive individuals as previously believed (Thomas et al., 2017). The competition between cell‐level selection favoring malignant, neoplastic cells, and organism‐level selection favoring individuals who are able to suppress mutations and rouge cells, has been central in the evolution of multicellularity, and accordingly, natural selection has shaped mechanisms that suppress cancer remarkably efficiently (Aktipis & Nesse, 2013). Since protective mechanisms against neoplastic cells are crucial for all multicellular organisms, some of these mechanisms are very old and well conserved, while others are unique and are shaped by the species’ lifestyle and ecology (Seluanov, Gladyshev, Vijg, & Gorbunova, 2018).

While studying model animals like mice and rats, which are highly susceptible to cancer, has led to great advances in our understanding of the process of tumorigenesis, this line of study has less to offer for understanding the mechanisms of cancer resistance (Seluanov et al., 2018). As a general rule, larger and longer‐lived animal species have more effective cancer suppression mechanisms (Caulin & Maley, 2011). In recent years, several discoveries providing novel insights on the natural mechanisms of cancer resistance have been made in nonstandard mammalian species, including naked mole rats (*Heterocephalus glaber,* Liang, Mele, Wu, Buffenstein, & Hornsby, 2010), blind mole rats (*Spalax ehrenbergi,* Gorbunova et al., 2012), long‐lived bats (Seim et al., 2013), whales (Keane et al., 2015), and elephants (Sulak et al., 2016). In each of these species, evolution has taken a different path, leading to novel mechanisms of cancer defense (Seluanov et al., 2018). Accordingly, studying a diversity of nonmodel species from the perspective of molecular mechanisms of natural cancer resistance can lead to new insights into the evolution of protective mechanisms against neoplastic cells and to a wider understanding of natural cancer defense mechanisms, which could eventually be harnessed for the development of human cancer therapies (Lemaître et al., 2019; Seluanov et al., 2018; Sepp, Ujvari, Ewald, Thomas, & Giraudeau, 2019).

So far, most of the studies of wild animal cancer defenses have focused on interspecific comparisons (but see Giraudeau et al., 2019), often looking at the duplication of specific known tumor suppressor genes. For example, it has been shown that elephants (*Loxodonta africana*) have more copies of tumor suppressor gene TP53 than other species (Sulak et al., 2016). Similarly, copy number gains involving genes associated with cancer have been shown in bowhead whales (*Balaena mysticetus*, Keane et al., 2015). Data for genomewide expression of aging‐associated or cancer‐related genes are also available for a few wild model species (i.e., wolves *Canis lupus*, Charruau et al., 2016, greater mouse‐eared bats *Myotis myotis,* Huang, Jebb, & Teeling, 2016; Huang et al., 2019). A comparison of greater mouse‐eared bats miRNA (microRNAs, regulators of gene expression) expression with that of humans, pigs, and cows revealed four upregulated cancer‐related genes, three of which likely function as tumor suppressors and one as a tumorigenesis promoter (Huang et al., 2016). In addition, a study comparing short‐lived and longer‐lived strains of the fish *Nothobranchius furzeri* revealed differential expressions of miRNAs related to known tumor suppressor genes (Baumgart et al., 2012).

Here, we apply an intraspecific approach for studying tumor suppression mechanisms in wild animals. Since age has been shown to be one of the most important risk factors for cancer in humans and captive animals (Rozhok & DeGregori, 2016), comparing cancer suppression mechanisms between young and old individuals from a wild long‐lived species could give a valuable insight into the mechanisms of age‐related cancer suppression preferred by natural selection. For example, a recent longitudinal study in greater mouse‐eared bats indicated that, several miRNAs acting as tumor suppressors were upregulated, while miRNAs promoting cell cycle or carcinogenesis were downregulated with age (Huang et al., 2019). From this perspective, seabirds are a promising, albeit currently completely ignored candidate for studying cancer defense mechanisms, as they have a longer maximum life span than expected from their body size and rates of energy metabolism (Holmes & Ottinger, 2003).

In this study, we used a known‐age breeding colony of common gull (*Larus canus*, maximal recorded life span 34 years) to study the links between age and cancer defenses at the genetic level. To gain insight into the key molecular mechanisms underlying cancer defenses, we characterized the transcriptome of birds and compared the results with databases of known human cancer‐related genes to assess whether cancer‐related genes are differently expressed between old and young gulls, and between males and females. Additionally, we analyzed whether the cancer‐related genes differently expressed in our analyses overlap with aging‐associated genes in other species, to understand whether the age‐related patterns of cancer resistance in gulls are more likely phylogenetically conserved or unique to long‐lived seabirds.

## 2 MATERIAL AND METHODS

### 2.1 Field methods

Samples were collected on the May 24, 2018, from a free‐living, known‐age breeding colony of common gulls located on Kakrarahu islet in Matsalu National Park on the west coast of Estonia (58°46' N, 23°26' E). This colony has been constantly monitored for over 40 years. All birds hatched on this islet are banded as chicks, so the exact age of the birds who return to the hatching colony to breed is known. In addition to a metal band, birds are also marked with a plastic band for ease of monitoring, and details of their breeding (i.e., partner choice, start of breeding, number of eggs) are recorded each year. Breeding birds are highly faithful to their colony, less than 3% of them change colonies between years, moving mostly to neighboring colonies (Rattiste, 2004). Male and female take turns in feeding and incubating throughout the day. For replacing damaged plastic bands, a subset of birds is caught every year from their nests using spring traps. To avoid nest abandoning, all birds are caught after the tenth day of incubation. From the birds needing to be caught for plastic band replacement, we chose 20 for collecting blood samples. All of these birds were caught on the same day, between 9 and 12 a.m., and released immediately after band change and blood sampling. Up to 50 μl of blood was collected from the brachial vein using blood lancets. Blood was collected in 200‐μl microvette tubes with EDTA as an anticoagulant, and about 10 μL of whole blood was then immediately transferred to RNAlater buffer. Samples were placed in a cooled and light‐protected box and transported to storage at −80ºC until analyzed. Ten birds were selected for transcriptome analysis, based on the extracted RNA quality, distribution of birds between age classes, and their gender (Table 1). Maximum life span of common gulls is 34 years, and about half of the birds survive over the age of 8–9 years (Rattiste & Lilleleht, 1986). The experimental protocol was approved by the Ministry of Rural Affairs of the Republic of Estonia (license no. 125, issued on May 22, 2018) and was performed in accordance with relevant Estonian and European guidelines and regulations.

**TABLE 1:** Demographic data of birds chosen for the transcriptome analysis

Age group	Hatch year	Breeding year	Sex
Young	2014	1st	M
Young	2014	2nd	M
Young	2014	2nd	M
Young	2015	1st	F
Young	2014	1st	F
Old	1989	27th	M
Old	Not known	24th	F
Old	2002	13th	M
Old	2002	13th	F
Old	2001	17th	M

### 2.2 Sequencing

Total RNA was extracted from RNALater preserved whole blood using RNeasy Mini Kit (Qiagen) according to manufacturer's instructions. The extraction also incorporated the optional DNAse digestion step. The initial quality and quantity of total RNA was determined using TapeStation (Agilent). On average 10 ± 3.4(*SD*) μg of total RNA was extracted with an average RIN 8.2 ± 2 (*SD*). The mRNA was extracted and cDNA generated using IlluminaTruSeq RNA Library Prep Kit v2. Paired end 95‐bp sequencing was performed on an Illumina HiSeq2500 sequencer (Sequencing kit: HiSeq Rapid PE Cluster Kit v2, Flowcell version: RapidRunV2) at the Institute of Genomics of Tartu University. The initial quality of the reads was then assessed using FastQC.

### 2.3 Transcriptome assembly and annotation

The sequencing resulted in 320M PE reads of 95 bp. Read cleaning and adapter trimming using Trimmomatic 0.38 (Bolger, Lohse, & Usadel, 2014) kept 99.5% of raw reads. Subsequent de novo transcriptome assembly was generated with Trinity 2.8.4 (Haas et al., 2013). Downstream analyses for aligning reads to assembly and differential expression were performed within Trinity using Salmon (Patro, Duggal, Love, Irizarry, & Kingsford, 2017) and edgeR (McCarthy, Chen, & Smyth, 2012), respectively. The edgeR package calculated false discovery rate (FDR) using Benjamini–Hochberg multiplicity correction method. The assembly quality was assessed by Benchmarking Universal Single‐Copy Orthologs BUSCO (Simão, Waterhouse, Ioannidis, Kriventseva, & Zdobnov, 2015). To annotate the obtained transcriptome, we used Dammit (Scott et al., 2019). We modified it to use the latest orthologous genes database (OrthoDB) version 10.0 (Kriventseva et al., 2019). The Dammit protocol comprises of several steps. First, TransDecoder version 5.5.0 (Haas et al., 2013) was used to determine open reading frames (ORFs). Then, the discovered protein domains were annotated using HMMER (Johnson, Eddy, & Portugaly, 2010) on the Pfam database (El‐Gebali et al., 2019). Finally, Dammit maps the transcripts to the OrthoDB and returns the best matching OrthoDB cluster ID. To identify the human orthologues, we took this OrthoDB cluster ID and then used it on the OrthoDB API to retrieve human orthologues for each transcript. The OthoDB API returned several possible human orthologue genes for each OrthoDB cluster ID. We included all genes returned from the OrthoDB API in further analyses irrespective of the reported mapping accuracy. The advantage of this approach is that all possible matches are found. The disadvantage is that some included matches are redundant. Human cancer‐related genes were obtained from the COSMIC cancer gene database (September 2019 version, Sondka et al., 2018) and joined with the OrthoDB. The joining was made using transcript human orthologues (human gene symbols and their synonyms) retrieved from the OrthoDB API and the gene symbols from the COSMIC cancer gene database. To check for OrthoDB version 10.0 redundant matches, the final list of cancer genes was checked manually using the OrthoDB version 10.1 website and matches that were removed between version changes were identified (listed in Table S3). To identify possible genes that vary with age, we used the list of mammal age‐related genes from the GenAge database (Magalhães & Toussaint, 2004). Custom database joins were performed using either SQLite version 3.24.0 (Hipp, Kennedy, & Mistachkin, 2018) or dplyr package in R version 3.5.3 (R Core Team, 2017). To determine the differentially expressed genes in the final dataset, we applied an FDR of 0.05 that was calculated based on all of the assembled transcripts not only using transcripts that were left in the dataset after joining the databases. Ggplot2 package (Wickham, 2009) was used for producing graphs. Source code of the updated version of Dammit protocol using the version 10 of OrthoDB can be accessed from (https://github.com/rix133/dammit/tree/OrthoDBv10). The raw sequencing data along with the assembled transcriptome are openly available in EMBL‐EBI European Nucleotide Archive under the primary study accession number PRJEB35479.

## 3 RESULTS

**FIGURE 1:**
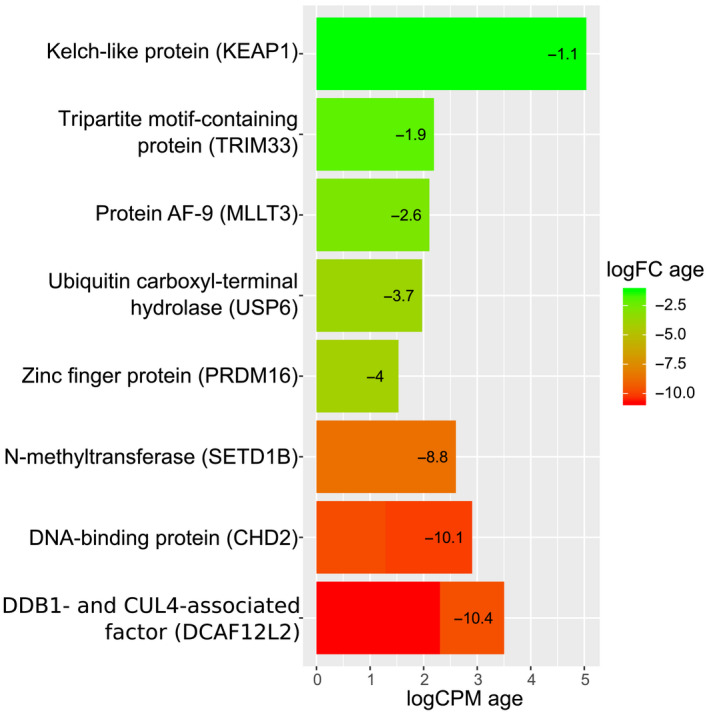
Cancer‐related differentially expressed genes between old and young common gulls. logCPM denotes the average log2‐counts‐per‐million transcripts (i.e., transcript abundance); logFC is log2 fold change between the groups. For example, value 4 means that the expression has increased 16‐fold. Y‐axis gene names are shortened names from the OrthoDB name; values in parentheses refer to the associated human gene symbols. See also Table S3 for detailed statistics

The Trinity de novo assembly resulted in 273,527 transcripts with a median length of 538 bp. The mean length of the assembled contigs was 1,686 bp (N50 = 4,133). Around 90% of vertebrate BUSCOs were present in the transcriptome assembly (C:81.7%[S:25.4%,D:56.3%],F:8.5%,M:9.8%,n:2,586). Dammit enabled to annotate 129,139 (~47%) contigs using OrthoDB. Differential expression analysis using edgeR retained 38 451 of those transcripts using default filtering values. 19,829 (Table S1) of those had a human orthologue in the OrthoDB and 3,305 (Table S2) could be matched to a gene in the COSMIC cancer gene database. After correcting for possible false positives, there were 39 differentially expressed transcripts by sex (9 matched genes, see Table S1) and 431 by age (220 matched genes, see Table S4). After removing the transcripts that had low abundance, no matches were found in the COSMIC database for sex‐related differentially expressed genes. Two hundred of genes differentially expressed between age groups were downregulated and 20 upregulated in old birds compared to the young birds. Thirty of the transcripts that displayed differential expression by age were also present in the COSMIC cancer gene database (Table S2). After removing transcripts that had low abundance (logCPM < 1) and grouping similar transcripts, this list was reduced to eleven cancer‐related genes (Table S3). Out of the eleven differentially expressed cancer‐related genes between young and old common gulls, three were likely artifactually linked to cancer after checking with latest OrthoDB version 10.1. The remaining eight were downregulated in old gulls compared to young ones. These eight transcripts were linked to the following cancer‐related genes: TRIM33, USP6, PRDM16, SETD1B, MLLT3, KEAP1, CHD2, and DCAF12L2 (Figure 1 and Table 2). The downregulation of the first five could be interpreted as a mechanism suppressing neoplasia risk and the downregulation of the last three as increasing the risk (see Table 2 for a description of the gene functions). We identified seven age‐related genes from the GenAge list that could be matched to one or several differentially regulated gull transcripts (see Table S4). These were as follows: JAK2, A2M, TFDP1, EGF, RICTOR, SIRT7, VCP. We found no overlap between the list of cancer‐associated and age‐related transcripts. Accordingly, none of the cancer‐related genes that were expressed differently between old and young gulls are known senescence genes in other studied species.

**TABLE 2:** The links of the transcripts that were differently expressed between young and old gulls with cancer‐related genes, and the possible function of these genes

Reduced OrthoDB name	COSMIC orthologue	Function of the gene	Hypothetical effect on neoplasia risk	References
Serine/threonine protein kinase (STK)	Tripartite motif‐containing 33, also known as transcriptional intermediary factor 1 gamma (TRIM33, TIF1‐γ)	Prevents apoptosis, has a variety of cellular functions, including cell growth, differentiation, immune response, and carcinogenesis.	Lower expression in old birds, which possibly lowers cancer risk	Wang et al. (2015), Lee (2018)
Ubiquitin‐specific proteases (USP)	USP6, also known as TRE17	Upregulation of USP6 leads to bone neoplasms, and this gene is therefore considered as an oncogene. While USP6 is a hominoid‐specific gene that was formed as result of a recent evolutionary fusion of the ancestor genes TBC1D3 to USP32, both of the ancestor genes have also been linked with oncogenic processes.	Lower expression in old birds, which possibly lowers cancer risk	Oliveira et al. (2004), Paulding, Ruvolo, and Haber (2003)
Zinc finger proteins (ZNFs)	PRDM16	Mainly functions in haematopoietic development, attenuates reactive oxygen species‐related stress, and represses tumor formation and/or progression, has anti‐apoptotic properties.	Lower expression in old birds, which possibly lowers cancer risk	Ivanochko et al. (2019), Zhu et al. (2016)
N‐methyltransferase	SETD1B	Has roles in multiple biological processes and has been shown to be overexpressed in several cancer types. SET1B also regulates another gene, SETD1A, which supports mitotic processes and consequentially, its knockdown induces senescence.	Lower expression in old birds, which possibly lowers cancer risk	Lee, Tate, You, and Skalnik (2007), Yang & Ernst, 2017, Chen et al. (2019), Tajima et al. (2019)
Kelch‐like (KLHL) gene family	Kelch‐like ECH‐associated protein 1 (KEAP1)	Controls the stability and accumulation of nuclear factor erythroid 2‐related factor 2 (NRF2), which controls genes important for cellular defense against oxidative stress. Inactivation of KEAP1 by reactive oxigene species (ROS) strongly induces NRF2, and this phenomenon is often observed in cancer cells, which can thus acquire malignancy.	Lower expression in old birds, which possibly increases cancer risk	Motohashi & Yamamoto (2007), Dhanoa, Cogliati, Satish, Bruford, and Friedman (2013), Taguchi and Yamamoto (2017)
YEATS	Myeloid/lymphoid leukemia (MLLT3)	MLLT3 transcription factor governing the self‐renewal of human haematopoietic stem cells. Regulator of epithelial cells orientation in a tissue.	Lower expression in old birds, which possibly decreases cancer risk	Calvanese et al. (2019), Haribaskar et al. (2009)
Chromodomain helicase DNA‐binding (CHD)	CHD2 and CHD4	CHD2 plays a critical role in development, hematopoiesis, and tumor suppression, by modulating DNA damage responses at the chromatin level. CHD4 is a modulator of cellular proliferation, senescence, and apoptosis.	Lower expression in old birds, which possibly increases cancer risk	Nagarajan et al. (2009), Mills (2017)
DDB1‐ and CUL4‐associated factor (DCAF) genes	DCAF12L2	These genes play critical roles in many cellular processes, including cell proliferation, survival, DNA repair, and genomic integrity. DCAF12L2 has been shown to be mutated in several human cancers.	Lower expression in old birds, which possibly increases cancer risk	Lee & Zhou, 2007, Liu et al. (2012), Gylfe et al. (2013)

Note

For their role in cancer, please see Appendix S1.

## 4 DISCUSSION

The well‐known Peto's paradox highlights the gaps in our current knowledge explaining why animals with larger bodies and longer life spans do not have higher incidence of cancers (Rozhok & DeGregori, 2016). While the paradox remains unresolved, most investigations focused on the evolution of intracellular mechanisms that reduce the risk of cell transformation (Rozhok & DeGregori, 2016). The current study applies a novel, intraspecific approach in a nonmodel wild animal and improves our understanding of the mechanisms of age‐related cancer risk and cancer suppression.

While there has been a push toward applying transcriptome methods in ornithological studies over the recent years (Jax, Wink, & Kraus, 2018), little is known about the age‐specific gene expression in birds. Age‐specific gene expression in follicles of Peking ducks (*Anas platyrhynchos*) indicated no clear pattern regarding expression levels in relation to age (Ren et al., 2019), while in great tits (*Parus major*), age classes were not associated with differential gene expression levels in blood and liver (Watson, Videvall, Andersson, & Isaksson, 2017). Conversely, in the present study, we found that more than 90% of the differentially expressed genes (including also transcripts that were not related to cancer) were downregulated in older birds. The average life expectancy for ducks is three years and for great tits 2–3 years, with maximal life span of twenty years for ducks and 15 years for great tits, accordingly, both of these species have much shorter life expectancies than common gulls. We can therefore hypothesize that the lower level on gene expression in the blood of old common gulls is related to a longer life span, slower pace of life and metabolism level necessary for reaching older age (Auer, Dick, Metcalfe, & Reznick, 2018). It should nonetheless be noted that the 220 genes differentially expressed between young and old common gulls comprise only about 0.57% of all the transcripts compared in our analysis.

Our analysis allowed the identification of 11 genes that could be linked to cancer and which were differently expressed between young and old common gulls. Our comparison of age groups was rather conservative, since the applied false discovery filtered out only the most clearly differentially expressed transcripts. Accordingly, we are confident in the biological relevance of the found differences. Out of these eleven genes, one was expressed in higher levels in older gulls, while for ten others, the expression was downregulated in the “old” group. This could indicate a reduced expression of these genes with increasing age, and/or that this lower expression is a prerequisite condition for reaching old age in common gulls. Given the cross‐sectional nature of our study, we cannot outrule the possibility that some birds in the “young” group will also reach old age (16+ years in our study). However, as only 17% of common gulls who start breeding at the study site reach the age of 16 (Rattiste & Lilleleht, 1986), we can suggest that the “old“ group consists of birds that are physiologically or genetically better adapted to reach old age.

Based on the known functions of these genes in humans, we divided the differently expressed genes into two groups: genes that decrease cancer risk, and genes that increase cancer risk (see the Discussion below). Additionally, three of the transcripts that were identified in our analyses as being similar to cancer‐related genes were more convincingly linked to other aging‐associated genes and the links with cancer genes in these cases are likely an artifact of our analysis. This is confirmed by the fact that the analysis where these links appeared was performed on OrthoDB version 10.0 while using the most recent OrthoDB version 10.1 did not exhibit these links. Therefore, instead of being linked to cancer‐related human orthologues suggested by the analysis, these three transcripts are more strongly associated with the following age‐related human orthologues. (a) ferritin gene (linked to CDK6), which could be linked back to age‐associated pathologies in humans (Touitou et al., 1985); (b) amyloid beta precursor A4 gene (linked to EXT1), which is widespread in the majority of vertebrate species that do not cease reproduction in senescence, and where selection pressure is maintained into old age (Moir & Tanzi, 2019), and (c) adenylate cyclase 3 (linked to PTEN), which is regulating fat accumulation and insulin levels in mammals (Liang et al., 2016). More details about the functions of these and also all the other transcripts are included in the Appendix S1.

The description of the functions of the eight remaining genes is presented in Table 2. The downregulation of 5 of these genes in older gulls could be a mechanism of cancer suppression. The first transcript could represent TRIM33 gene (tripartite motif‐containing 33, also TIF1‐γ), a transcriptional cofactor that prevents apoptosis (Wang et al., 2015), and similar role could be ascribed to the second transcript linked to PRDM16 gene (Zhu et al., 2016). Downregulation of these genes in older gulls could stimulate apoptosis and thus limit cancer progression. The third transcript was linked to USP6, which, when upregulated, is considered an oncogene causing bone neoplasms (Oliveira et al., 2004), and downregulation of this gene in older gulls suggests an anticancer mechanism. The fourth transcript was linked to SETD1B, which supports mitotic processes (Tajima et al., 2019) and is overexpressed in several cancer types (e.g., Yang & Ernst, 2017; Chen et al., 2019). A lower expression of SETD1B in older gulls thus suggests a method for suppressing uncontrolled cell growth and thus neoplasia. The fifth transcript was linked to MLLT3 (protein AF‐9), which has been associated with leukemia in several vertebrate species (Ney Garcia et al., 2015). While its upregulation enables cell proliferation (Calvanese et al., 2019), downregulation reduces it (Zhang, Luo, Wang, & Yang, 2012). Hence, the downregulation of MMLT3 in common gull blood cells with age might reflect increased cancer resistance. On the other hand, the 3 other genes that were downregulated in older birds could potentially lead to increased cancer risk. The first transcript was linked to KEAP1 (Kelch‐like ECH‐associated protein 1), which, through its link to oxidative stress regulating genes, such as glutamate–cysteine ligase and glutathione reductase (Yang et al., 2013), is considered as a tumor suppressor gene. Interestingly, our previous studies in common gulls have indicated that the glutathione system is indeed linked to the longevity of these birds (Urvik et al., 2016). Lower expression of a tumor suppressor in older gulls could increase cancer risk. The second transcript was linked to the gene DCAF12L2, which was shown to be mutated in several human cancers (Gylfe et al., 2013; Liu et al., 2012). However, the role of this gene in human cancers is still under investigation. The third transcript was associated with CHD2 and CHD4 genes, which play a critical role in tumor suppression (Nagarajan et al., 2009), but also in cellular proliferation, senescence, and apoptosis (Mills, 2017). Again, a lower expression of tumor suppressor genes in older gulls could suggest higher cancer risk.

**FIGURE 2:**
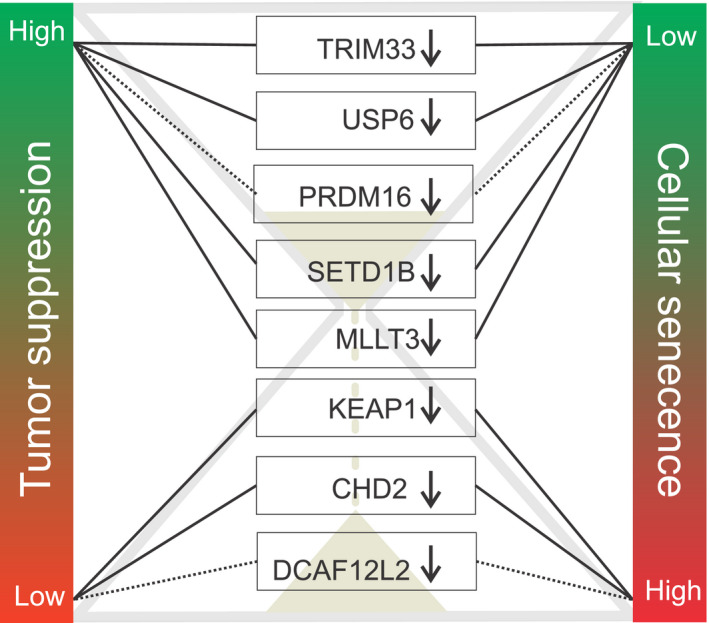
Eight cancer‐related genes that had lower expression in old common gulls (>16 years old) compared to young ones (3–4 years old), and the possible links of this downregulation with tumor suppression and neoplasia risk. Dashed lines indicate less certainty in depicted associations, according to published literature

To summarize these results, out of eleven differentially expressed cancer‐related genes between young and old common gulls, three were most likely artifactually linked to cancer, since these transcripts are associated with aging processes through more straightforward physiological mechanisms. Out of the eight remaining genes, the downregulation of five in old birds could be interpreted as a tumor suppressor mechanism, and three as potentially tumor promoting (Figure 2). Since cancer (and cancer suppression mechanisms) is an evolutionarily very old issue (Nesse, 2017), we can expect many of the genetic mechanisms related to cancer to be phylogenetically conserved. This was also apparent in our results, as most of the differently expressed cancer‐related genes appeared to be well conserved. This phylogenetic conservation allows the use of human orthologues for interpreting the results. Nevertheless, we wish to stress that this interpretation should be made with caution. Indeed, most of the existing knowledge about the links between specific genes and cancer susceptibility comes from either studies of genetic mutations in human cancer patients or experiments with knockout mice. Such a knowledge in wild animal species is thus virtually nonexistent. Since differences between species (and, moreover, vertebrate classes) are frequent, it is still difficult to accurately interpret differences in cancer‐related gene expression levels in the context of higher or lower cancer risk.

May the observed age‐specific patterns in gene expression be influenced by the physiological changes associated with incubation? As we cannot capture the same birds outside the reproductive period or even during other stages of breeding/incubation (to avoid nest abandonment), we cannot outrule the possibility that the observed pattern in cancer gene expression is only present during this life stage. It is possible that old and young birds respond differently to this physiologically demanding period. Our previous studies have shown, for example, that older birds deposit less antioxidants in their eggs than younger birds, possibly with the aim of maintaining higher antioxidant level within their own (aging) body (Urvik et al., 2018). As has been suggested before (Aktipis, Boddy, Gatenby, Brown, & Maley, 2013), the links between (the cost of) reproduction, aging, and cancer probability are very likely. From this viewpoint, sampling birds during the incubation period is probably the best strategy for revealing these life‐history trade‐offs. To understand these associations in the context of seasonal changes in bird physiology, it would be very necessary to capture the same birds during other seasons; however, with this model system, it would be very challenging.

Knowledge about cancer prevalence and cancer types in common gulls is an essential next step for establishing this species as a model organism for cancer research. As of now, the research on wildlife cancer is very limited due to lack of easily applicable methodology. In closely related species, silver gulls (*Larus novaehollandiae*), lymphocytic lymphomas and mixed‐cell lymphomas have been described, and chondromas and myelocytomas have been described in pacific gulls (*Larus pacificus*) (Ladds, 2009; Reece, 1992). It is still not known how strong selection pressure cancer could be for gulls. What we do know, based on studies in common gulls, is that on the last year of life, their reproductive success will drop (Rattiste, 2004), suggesting that the death of gulls is not only random predation, but that physiological senescence plays a role. Future effort should be targeted at trying to determine the cause of mortality in common gulls, for example, by collecting carcasses and performing necropsy. Even species with very low cancer prevalence have to invest in cancer prevention, possibly even more than species with high cancer prevalence, making them intriguing models for understanding the evolutionary mechanisms of cancer defense (Thomas et al., 2017). Common gulls might have genetic mechanisms for suppressing cancer that allow these birds to bypass some of the processes linked to senescence, allowing them to reach old age without increased risk of cancer. This is further supported by the finding that none of the identified cancer‐related genes expressed differently between age groups has been associated directly with aging in humans or model species. We did identify seven differently expressed transcripts that could be linked with known human aging‐associated genes as curated in the GenAge database, but there was no overlap with cancer‐related genes revealed by our analysis. These results suggest that cancer suppression might represent an evolutionary adaptation that enables these seabirds to achieve longevity, despite their high metabolic rate and small body size. For example, when looking at telomere shortening, which is considered a mechanisms mediating trade‐offs between senescence and cancer susceptibility (Nesse, 2017), previous studies indicated that unlike many other species, older gulls do not have shorter telomeres when compared to younger gulls (Rattiste et al., 2015). Early in life, in the fastest growth phase, their telomeres might actually elongate instead of shortening (unpublished data). Our results therefore call for further investigations focused on (a) the comparison of gene expression between common gulls that exhibit signs of cancer or not (although cancer diagnostics in wild animals need to take a step forward), and (b) the comparison of our results with data from other long‐lived seabirds, in order to evaluate if the found patterns in cancer‐related genes are universal among bird species with similar life histories.

In conclusion, we have shown that old gulls differ from young ones both from the aspect of cancer susceptibility and tumor suppression at the genetic level. This is the first study to look at intraspecific variations in cancer defenses in relation to aging at the genetic level in a wild bird species. It is intriguing to speculate that these seabirds have, through evolutionary pressure for a longer life span, found physiological or genetic pathways to bypass the inevitable process of senescence (see also Rattiste et al., 2015; Urvik et al., 2016). Hopefully, future years will bring a fast accumulation of data on the genetic mechanisms of cancer defenses in nonmodel species, thereby improving our understanding of this phylogenetically very old, but at the same time very contemporary issue.

## CONFLICT OF INTEREST

None declared.

## Supporting information

Appendix S1

Table S1‐S4

## Data Availability

The raw sequence reads and the transcriptome assembly are made available in the European Nucleotide Archive under the study accession no. PRJEB35479. Rest of the additional data can be found from the Appendix S1.
