# Supplementary material for: Age‐dependent expression of cancer‐related genes in a long‐lived seabird
Source: Evol Appl. 2020 Jun 15;13(7):1708–18. doi: 10.1111/eva.13024 (PMC7428815; doi:10.1111/eva.13024)
Supplement: Supplementary file 1 — Appendix S1 [file EVA-13-1708-s001.docx]

Supplementary Materials for the article

**Age-dependent expression of cancer-related genes in a long-lived seabird**

**Detailed description of genes related to cancer in our data set**

The only cancer-related gene that was significantly more expressed in older birds was related to ferritin (Figure 1), which is a universal iron storage protein, and thereby has a role in preventing reactive oxygen species (ROS) production by free iron (DeRuisseau et al. 2013). A strict regulation of iron storage is essential to maintain cellular homeostasis and integrity and,in humans, serum/plasma ferritin concentration correlates closely with body iron stores. However, since ferritin is an acute phase protein, it is also elevated during infection or inflammation (Fairweather-Tait et al. 2014). It is therefore suggested that the repeatedly reported increase of ferritin in the aged human populations is merely related to an age-associated pathology and may not be a normal physiological event occurring during the process of aging (Toitou et al. 1985). Since the role of ferritin in avian senescence has not been studied so far, we cannot conclude if the high levels of ferritin in older birds are related to oxidative stress prevention or age-related pathology. While ferritin has been linked with many pathways related with cancer, such as cell proliferation, growth suppressor evasion, cell death inhibition, immortalization, angiogenesis, invasion and metastasis, and immunomodification (reviewed in Min Pang and Connor, 2015), its role as an oncogene or tumor suppressor gene is questionable. In our study, it gave a signal as a cancer-related gene only through the human orthologues database (OrthoDB) and COSMIC cancer gene database, due to similarities in relevant catalythical sequences with the cycline-dependent kinase 6. However, the difference of our sequence from the consensus sequence was more than two standard deviations. Cyclin-dependent kinases (CDKs) are a family of serine/threonine protein kinases that are involved in the cell cycle, transcription and other biological processes such as translation, neurogenesis and apoptosis. CDK6 is known to play a role in overcoming aspects of replicative senescence, whether induced by telomere dysfunction or proliferative stress (Ruas et al. 2007). Deregulation of CDKs is directly linked to oncogenesis (Tadesse et al. 2015). While higher CDK6 levels in the blood of older birds would suggest higher vulnerability to cancer, it is more likely that this link with CDK6 in our study is artefactual, and this gene transcript should be instead interpreted as indicating higher ferritin levels in older birds.

Another gene that was diffrently expressed between old and young gulls is the amyloid beta A4 precursor gene (i.e. down regulation in old birds) which code amyloid precursor protein (APP), an ancient and highly conserved protein (Tharp and Sarkar, 2013). APP has been suggested to regulate synapse formation, neural plasticity, antimicrobial activity, and iron export, though its primary function is not known (Duce et al. 2010, Moir and Tanzi, 2019). While in humans, APP is mainly considered a pathological protein that has persisted in human genome due to the negative effects appearing only in post-reproductive age, it is also widespread in the majority of vertebrate species that do not cease reproduction in senescence, and where selection pressure is maintained into old age (Moir and Tanzi, 2019). The link between this gene and cancer in birds is however questionable, and might be an artefactual result of mapping of the transcript with human orthologues. This method indicated a similarity of the transcript to exostosin glycosyltransferase 1 gene (EXT1), and mutations of this gene have been related to higher risk of bone cancer in humans (Hameetman et al. 2004). EXT1 is therefore considered a tumor suppressor. However, a link between APP gene and EXT1 gene has not been described in scientific literature, accordingly, this result is more likely to be interpreted as a downregulation of APP production in older birds.

We found a lower expression of a transcription factor Protein AF-9 (MLLT3) in older gulls, MLLT3 (Chromatin reader component of the super elongation complex) has been associated with myeloid/lymhoid leukemia in humans, but also in other vertebrates (Ney Garcia et al. 2015). While its upregulation enables hematopoietic cell proliferation (Calvanese et al. 2019), downregulation reduces it (Zhang et al 2012). MLLT3 also takes part in planar cell polarity regulation ([Haribaskar](https://www.sciencedirect.com/science/article/pii/S0006291X09013461?via%3Dihub#!), et al. 2009). Maintaining proper cell polarity regulation is key to longevity (Budovsky et. al 2010). Hence, the downregulation of MMLT3 in common gull blood cells with age might reflect increased cancer resistance but may also be a age related decline in cell polarity regulation.

We found a lower expression level of the serine/threonine protein kinases (STK) in older birds. STK-s play a crucial role in cellular homeostasis and signaling through their ability to phosphorylate transcription factors, cell cycle regulators, and a vast array of cytoplasmic and nuclear effectors (Edelman et al. 1987). In humans, several types of STK-s are overexpressed in different cancers, but display a low or absent expression in normal tissues, which suggests a putative tumor suppressor role (Capra et al. 2006). The human orthologue of this transcript, the tripartite motif-containing 33 (TRIM33, also known as transcriptional intermediary factor 1 gamma (TIF1-γ)) is a transcriptional cofactor that prevents apoptosis (Wang et al. 2015). The TRIM family of proteins is a highly conserved group of proteins that have a variety of cellular functions, including cell growth, differentiation, immune response, and carcinogenesis (Lee, 2018). In mice hematopoietic stem cells, deletion of TRIM33 resulted with an accelerated aging phenotype. The lower expression in older birds measured here therefore suggest a (compensatory?) mechanism for controlling cell proliferation and thus limit cancer progression, or could be an inevitable symptom of advanced age.

Ubiquitin-specific proteases (USP) remove a small regulatory protein, ubiquitin, from larger proteins and thereby affect their cellular localization, acitivation and degradation (Wilkinson, 1999). Loss of regulation by protein ubiquitination leads to various diseases, including cancer (Young et al. 2019). USP expression is markedly increased in several malignant tumors (Yuan et al. 2018). Our search of similar cancer-related sequences suggested a human orthologue gene, USP6. Up-regulation of USP6 (also known as TRE17) transcription may lead to benign and malignant bone neoplasms, and this gene is therefore considered as an oncogen (Oliveira et al. 2004). While USP6 is a hominoid-specific gene that was formed as result of a recent evolutionary fusion of the ancestor genes TBC1D3 to USP32 (Paulding et al, 2003), both of the ancestor genes have also been linked with oncogenic processes (Oliveira et al. 2004). The role of USP-s in aging process is not known. We could therefore speculate that a decrease in USP production in older gulls could be a mechanism that prevents the loss of regulation of proteins and thereby also decreases the risk of tumorigenesis.

Zinc-finger proteins (ZNFs) are one of the most abundant groups of proteins and have a wide range of molecular functions, including transcriptional regulation, ubiquitin-mediated protein degradation, signal transduction, actin targeting, DNA repair, cell migration, and numerous other processes (Cassandri et al. 2017). ZNFs are involved in tumorigenesis, cancer progression and metastasis formation, and may act both as oncogenes or tumor suppressor genes (Cassandri et al. 2017). The human orthologue of our transcript, PRDM16 gene, mainly functions in haematopoietic development, attenuates reactive oxygen species-related stress, and can function to repress certain aspects of tumor formation and/or progression (Ivanochko et al. 2019). In the context of senescence, PRDM16 also acts as a transcriptional coregulator that controls the development of brown adipose tissue formation, which is known to decrease with advanced age (Harms et al. 2014). However, since only mammals possess brown adipose tissue, this role of PRDM16 could not be extrapolated to birds. Since PRDM16 has also been shown to have anti-apoptotic properties (Zhu et al. 2016), we could speculate that a reduced expression of this gene in older gulls might act as a mechanism of tumor suppression.

N-methyltransferase is an enzyme that catalyzes the N-methylation of various amino-acids. The gene corresponding to the sequence in our gull sample corresponds to the gene SETD1B, which is an important component of the histone methyltransferase complex that generates trimethylated histone H3 at Lys4 and has been implicated in multiple biological processes (Lee et al. 2007). In several cancer types, SETD1B has been shown to be overexpressed (e.g. Yang & Ernst, 2017; Chen et al. 2019). SET1B also regulates another gene, SETD1A, which supports mitotic processes and consequentially, its knockdown induces senescence (Tajima et al. 2019). A lower expression of SETD1B in older gulls therefore suggest a method for suppressing uncontrolled cell growth and thus neoplasia?.

The Kelch-like (KLHL) gene family is a phylogenetically conserved group of genes including Kelch-like ECH-associated protein 1 (KEAP1), which in our study was expressed in lower levels in older birds. KEAP1 controls the stability and accumulation of nuclear factor erythroid 2-related factor 2 (NRF2), which is a transcription factor that controls genes important for cellular defense against oxidative stress (Motohashi and Yamamoto 2007, Dhanoa et al. 2013). In normal, unstressed conditions, the cellular NRF2 level is very low, but it is dramatically increased upon exposure to ROS (Itoh 1997). Inactivation of KEAP1 by ROS strongly induces NRF2, and this phenomenon is often observed in cancer cells, which can thus acquire malignancy by perverting NRF2 activity, and thereby acquiring strong antioxidative activities (Taguchi and Yamamoto, 2017). KEAP1 is therefore considered as a tumor suppressor gene. In the context of cellular senescence, inactivation of KEAP1 has been shown to stimulate mRNA expression of Nrf2-targeted downstream genes with antioxidant effects, such as glutamate–cysteine ligase and glutathione reductase (Yang et al. 2013). Interestingly, our previous studies in common gulls have indicated that the glutathione system is indeed linked to the longevity of these birds (Urvik et al. 2016).

Adenylate cyclases are enzymes with with key regulatory roles in essentially all cells, catalysing the conversion of adenosine triphosphate (ATP) to 3',5'-cyclic AMP (cAMP) and pyrophosphate (Zhang et al. 1997). The cancer-related human orthologue of the differentially expressed gull sequence was phosphatase and tensin homolog (PTEN), which is one of the most well-known and evolutionarily conserved tumor suppressor genes (Pomerantz & Blau, 2013). PTEN deficiency, and the consequent overactivation of the phosphatidylinositol-3-kinase (PI-3) pathway, results in a hyperproliferative state and increased cellular survival (Pomerantz & Blau, 2013). PTEN also promotes another tumor suppressor, p53 activity (Pomerantz & Blau, 2013). We could therefore speculate that lower level of Pten expression in older gulls is again a mechanism that helps to slow down senescence with a possible cost of increased cancer risk. However, our link with PTEN was likely artefactual as this only appeared with the OrthoDB version 10.0 while this link was not present in the version 10.1 of the OrthoDB. Lower level of PTEN expression in older gulls could not necessarily mean increased cancer risk, since it has been shown that the loss of PTEN sometimes also elicits a senescence response (by activating tumor suppressor p53) that opposes tumorigenesis (Alimonti et al. 2010). Accordingly, tumor suppressors can differentially influence senescence, with too little or too much activity acting cytostatically (Alimonti et al. 2010). The actual right link to Adenylate cyclase 3 (ADCY3) may also related to cancer through regulating the cAMP-responsive element-binding pathway (Hong et al. 2013).

Chromodomain helicase DNA-binding (CHD) proteins have been identified as critical regulators of cellular processes such as stem cell quiescence, proliferation, and cell fate determination (Micucci et al. 2015). These ATP-dependent chromatin remodelers govern the cellular machinery’s access to DNA, thereby controlling fundamental processes, including transcription, proliferation, and DNA damage repair, and an inactivation of the CHD family of proteins therefore promotes tumorigenesis (Mills et al. 2017). From CHD genes, our analyses revealed different expression levels of CHD2 and CHD4 genes between old and young gulls. Studies in Chd2 heterozygous mutant mice have indicated that CHD2 protein appears to play a critical role in development, hematopoiesis, and tumor suppression, by modulating DNA damage responses at the chromatin level (Nagarajan et al. 2009). Chd2 mutants exhibited lowered capacity of erythrocyte differentiation, and later in life, Chd2 deficiency led to lymphomas (Nagarajan et al. 2009). Indeed, also in humans, CHD2 is one of the most frequently mutated genes in chronic lymphocytic leukemia (Rodríguez et al. 2015). CHD4 belongs to a subfamily of CHD proteins that includes potent modulators of cellular proliferation, senescence, and apoptosis (Mills, 2017). Lower expression levels of these genes in older gulls could therefore indicate again a preventive mechanism of senescence that could increase cancer risk.

DDB1- and CUL4-associated factor (DCAF) genes encode substrate receptor proteins for ubiquitin ligases, which play critical roles in many cellular processes, including cell proliferation, survival, DNA repair, and genomic integrity (Lee & Chow, 2007). Whether a higher DCAF gene expression level hastens or hinders oncogenic processes seems to depend on the specific type of DCAF (Yan et al. 2017). The human orthologue of the differentially expressed gull gene was DCAF12L2, which has been shown to be mutated in several human cancers and is therefore considered a candidate oncogene (Liu et al. 2012, Gylfe et al. 2013). Since the role of this gene in human cancers is still under investigation, it is early to speculate why DCAF12L2 is expressed in a lower level in older gulls and whether lower expression of DCAF12L2 might be associated with lower cancer risk in gulls. What we can say is that this gene is not a well-known tumor suppressor and, based on human studies, might be considered an oncogene.

**References**

Alimonti, A., Carracedo, A., Clohessy, J.G., Trotman, L.C., Nardella, C., Egia, A., … Pandolfi, P.P. (2010) Subtle variations in Pten dose determine cancer susceptibility. *Nature Genetics*, 42, 454-458.

Calvanese, V., Nguyen, A. T., Bolan, T. J., Vavilina, A., Su, T., Lee, L. K., Wang, Y., Lay, F. D., Magnusson, M., Crooks, G. M., Kurdistani, S. K., & Mikkola, H. K. A. (2019). MLLT3 governs human haematopoietic stem-cell self-renewal and engraftment. *Nature*, *576*(7786), 281–286.

Capra, M., Nuciforo, P.G., Confalonieri, S., Quarto, M., Bianchi, M., Nebuloni, M., ..., Di Fiore, P.P. (2006). Frequent alterations in the expression of serine/threonine kinases in human cancers. *Cancer Research,* 66, 8147-8154.

Cassandri, M., Smirnov, A., Novelli, F., Pitolli, C., Agostini, M., Malewicz, M., ..., Raschellà, G. (2017). Zinc-finger proteins in health and disease. *Cell Death Discovery,* 3, 17071.

Chen, D., Li, T., Wang, C., Lei, G., Wang, R., Wang, Z., … Yang, P. (2019). High‑level SETD1B gene expression is associated with unfavorable prognosis in hepatocellular carcinoma. *Molecular medicine reports*, 19, 1587-1594.

DeRuisseau, K.C., Park, Y.M., DeRuisseau, L.R., Cowley, P.M., Fazen, C.H., Doyle, R.P. (2013). Aging-related changes in the iron status of skeletal muscle. *Experimental gerontology*, 48, 1294–1302.

Duce, J.A., Tsatsanis, A., Cater, M.A., James, S.A., Robb, E., Wikhe, K., ..., Bush, A.I. (2010). Iron-export ferroxidase activity of β-amyloid precursor protein is inhibited by zinc in Alzheimer's disease. *Cell,* 142, 857–867.

Edelman, A.M., Blumenthal, D.K., Krebs, E.G. (1987). Protein serine/threonine kinases. *Annual Review of Biochemistry*, 56, 567–613.

Fairweather-Tait, S.J., Wawer, A.A., Gillings, R., Jennings, A., Myint, P.K. (2014). Iron status in the elderly. *Mechanisms of ageing and development*, 136-137, 22–28.

Gylfe, A.E., Kondelin, J., Turunen, M., Ristolainen, H., Katainen, R., Pitkänen, E., ... Aaltonen, L.A. (2013). Identification of candidate oncogenes in human colorectal cancers with microsatellite instability. *Gastroenterology*, 145, 540-543.

Hameetman, L., Bovée, J.V., Taminiau, A.H., Kroon, H.M., Hogendoorn, P.C. (2004). Multiple osteochondromas: clinicopathological and genetic spectrum and suggestions for clinical management. *Hereditary cancer in clinical practice*, 2, 161–173.

Haribaskar, R., Pütz, M., Schupp, B., Skouloudaki, K., Bietenbeck, A., Walz, G., & Schäfer, T. (2009). The planar cell polarity (PCP) protein Diversin translocates to the nucleus to interact with the transcription factor AF9. *Biochemical and Biophysical Research Communications*, *387*(1), 212–217.

Harms, M., Ishibashi, J., Wang, W., Lim, H.W., Goyama, S., Sato, T., ..., Seale, P. (2014) Prdm16 is required for the maintenance of brown adipocyte identity and function in adult mice. *Cell Metabolism*, 9, 593–604.

Hong, S.-H., Goh, S.-H., Lee, S. J., Hwang, J.-A., Lee, J., Choi, I.-J., Seo, H., Park, J.-H., Suzuki, H., Yamamoto, E., Kim, I.-H., Jeong, J. S., Ju, M. H., Lee, D.-H., & Lee, Y.-S. (2013). Upregulation of adenylate cyclase 3 (ADCY3) increases the tumorigenic potential of cells by activating the CREB pathway. *Oncotarget*, *4*(10), 1791–1803.

Itoh, K., Chiba, T., Takahashi, S., Ishii, T., Igarashi, K., Katoh, Y., ..., Nabeshima, Y. (1997). An Nrf2/small Maf heterodimer mediates the induction of phase II detoxifying enzyme genes through antioxidant response elements. *Biochemical and Biophysical Research Communications*, 236, 313-322.

Ivanochko, D., Halabelian, L., Henderson, E., Savitsky, P., Jain, H., Marcon, E., … Arrowsmith, C.H. (2019). Direct interaction between the PRDM3 and PRDM16 tumor suppressors and the NuRD chromatin remodeling complex. *Nucleic Acids Research*, 47, 1225-1238.

Lee, J., Zhou, P. (2007) DCAFs, the missing link of the CUL4-DDB1 ubiquitin ligase. *Molecular cell*, 26, 775-780.

Lee H.J. (2018). The role of tripartite motif family proteins in TGF-β signaling pathway and cancer. *Journal of Cancer Prevention,* 23, 162–169.

Liu, P., Morrison, C., Wang, L., Xiong, D., Vedell, P., Cui, P., … You, M. (2012). Identification of somatic mutations in non-small cell lung carcinomas using whole-exome sequencing. *Carcinogenesis*, 33, 1270–1276.

Micucci, J.A., Sperry, E.D., Martin, D.M. (2015). Chromodomain helicase DNA-binding proteins in stem cells and human developmental diseases. *Stem Cells and Development*, 24, 917–926.

Mills A.A. 2017. The chromodomain helicase DNA-binding chromatin remodelers: family traits that protect from and promote cancer. *Cold Spring Harbor Perspectives in Medicine*, 7, a026450.

Min Pang, B.S., Connor, J.R. (2015) Role of ferritin in cancer biology. *Journal of Cancer Science & Therapy* 7, 155-160.

Moir, R.D., Tanzi, R.E. (2019). Low evolutionary selection pressure in senescence does not explain the persistence of Aβ in the vertebrate genome. *Frontiers in Aging Neuroscience*, 11, 70.

Motohashi, H., Yamamoto, M. (2007). Carcinogenesis and transcriptional regulation through Maf recognition elements. *Cancer Science*, 98, 135-139.

Nagarajan, P., Onami, T.M., Rajagopalan, S., Kania, S., Donnell, R., Venkatachalam, S. (2009). Role of chromodomain helicase DNA-binding protein 2 in DNA damage response signaling and tumorigenesis. *Oncogene*, 28, 1053-1062.

Ney Garcia, D.R., Liehr, T., Emerenciano, M., Meyer, C., Marschalek, R., Pombo-de-Oliveira, M.D.S., (...), Macedo Silva, M.L. (2015). Molecular studies reveal a MLL-MLLT3 gene fusion displaced in a case of childhood acute lymphoblastic leukemia with complex karyotype. *Cancer Genetics*, 208, 143-147.

Oliveira, A.M., Perez-Atayde, A.R., Inwards, C.Y., Medeiros, F., Derr, V., Hsi, B.L., … Fletcher, J.A. (2004). USP6 and CDH11 oncogenes identify the neoplastic cell in primary aneurysmal bone cysts and are absent in so-called secondary aneurysmal bone cysts. *The American Journal of Pathology*, 165, 1773–1780.

Paulding, C.A., Ruvolo, M., Haber, D.A. (2003). The Tre2 (USP6) oncogene is a hominoid-specific gene. *Proceedings of the National Academy of Sciences USA*, 100, 2507-2511.

Pomerantz, J.H., Blau, H.M. (2013) Tumor suppressors: enhancers or suppressors of regeneration? *Development* 140, 2502-2512.

Rodríguez, D., Bretones, G., Quesada, V., Villamor, N., Arango, J.R., López-Guillermo, A., ..., López-Otín, C. (2015) Mutations in CHD2 cause defective association with active chromatin in chronic lymphocytic leukemia. *Blood,* 126, 195-202.

Ruas, M., Gregory, F., Jones, R., Poolman, R., Starborg, M., Rowe, J., … Peters, G. (2007). CDK4 and CDK6 delay senescence by kinase-dependent and p16INK4a-independent mechanisms. *Molecular and cellular biology*, 27, 4273–4282.

Tadesse, S., Yu, M., Kumarasiri, M., Le, B.T., Wang, S. (2015). Targeting CDK6 in cancer: State of the art and new insights. *Cell Cycle*, 14, 3220–3230.

Taguchi, K., Yamamoto, M. (2017). The KEAP1-NRF2 System in Cancer. *Frontiers in Oncology*, 7, 85.

Tajima, K., Matsuda, S., Yae, T., Drapkin, B.J., Morris, R., Boukhali, M.,… Maheswaran, S. (2019). SETD1A protects from senescence through regulation of the mitotic gene expression program. *Nature Communications*, 10, 2854.

Tharp, W.G., Sarkar, I.N. (2013). Origins of amyloid-β. *BMC genomics*, 14, 290.

Touitou, Y., Proust, J., Carayon, A., Klinger, E., Nakache, J-B., Huard, D., Sachet, A. (1985). Plasma ferritin in old age. Influence of biological and pathological factors in a large elderly population. *Clinica Chimica Acta,* 149, 37-45.

Urvik, J., Meitern, R., Rattiste, K., Saks, L., Hõrak, P., Sepp, T. (2016) Variation in the markers of nutritional and oxidative state in a long-lived seabird: associations with age and longevity. *Physiological and Biochemical Zoology*, 89, 417-440.

Wang, E., Kawaoka, S., Roe, J. S., Shi, J., Hohmann, A. F., Xu, Y., … Vakoc, C. R. (2015). The transcriptional cofactor TRIM33 prevents apoptosis in B lymphoblastic leukemia by deactivating a single enhancer. *eLife*, 4, e06377.

Wilkinson, K.D. (1997). Regulation of ubiquitin-dependent processes by deubiquitinating enzymes. *FASEB Journal*, 11, 1245–1256.

Yan, H., Bi, L., Wang, Y., Zhang, X., Hou, Z., Wang, Q., ..., Mao, J.-H. (2017) Integrative analysis of multi-omics data reveals distinct impacts of DDB1-CUL4 associated factors in human lung adenocarcinomas. *Scientific Reports,* 7, 333.

Yang, W., Ernst, P. (2017). Distinct functions of histone H3, lysine 4 methyltransferases in normal and malignant hematopoiesis. *Current Opinion in Hematology*, 24, 322–328.

Yang, G., Zhao, K., Ju, Y., Mani, S., Cao, Q., Puukila, S., ... Wang, R. (2013) Hydrogen sulfide protects against cellular senescence via S-sulfhydration of Keap1 and activation of Nrf2. *Antioxidants & Redox Signaling*, 20, 1906-1919.

Young, M.-R., Hsu, K.-C, Lin, T.E., Chang, W.-C., Hung, J.-J. (2019). The role of ubiquitin-specific peptidases in cancer progression. *Journal of Biomedical Science*, 26, 42.

Yuan, T., Yan, F., Ying, M., Cao, J., He, Q., Zhu, H., Yang, B. (2018). Inhibition of ubiquitin-specific proteases as a novel anticancer therapeutic strategy. *Frontiers in Pharmacology*, 9, 1080.

Zhang, G., Liu, Y., Ruoho, A.E., Hurley, J.H. (1997) Structure of the adenylyl cyclase catalytic core. *Nature,* 386, 247–253.

Zhang, T., Luo, Y., Wang, T., & Yang, J. Y. (2012). MicroRNA-297b-5p/3p target Mllt3/Af9 to suppress lymphoma cell proliferation, migration and invasion in vitro and tumor growth in nude mice. *Leukemia & Lymphoma*, *53*(10), 2033–2040.

Zhu, S., Xu, Y., Song, M., Chen, G., Wang, H., Zhao, Y., ... Li, F. (2016) PRDM16 is associated with evasion of apoptosis by prostatic cancer cells according to RNA interference screening. *Molecular Medicine Reports*, 14, 3357‐3361.
